# Anatomic Risk Factors for Initial and Secondary Noncontact Anterior Cruciate Ligament Injury: A Prospective Cohort Study in 880 Female Elite Handball and Soccer Players

**DOI:** 10.1177/03635465241292755

**Published:** 2024-11-18

**Authors:** Yusuke Kamatsuki, Marie Synnøve Qvale, Kathrin Steffen, Arnlaug Wangensteen, Tron Krosshaug

**Affiliations:** † Oslo Sports Trauma Research Center, Department of Sports Medicine, Norwegian School of Sport Sciences, Oslo, Norway; Investigation performed at the Department of Sports Medicine, Norwegian School of Sport Sciences, Oslo, Norway

**Keywords:** female, soccer, handball, anterior cruciate ligament, knee valgus, hyperextension

## Abstract

**Background:**

Anterior cruciate ligament (ACL) injury is one of the most severe injuries for athletes. It is important to identify risk factors because a better understanding of injury causation can help inform athletes about risk and increase their understanding of and motivation for injury prevention.

**Purpose:**

To investigate the relationship between anatomic factors and risk for future noncontact ACL injuries.

**Study Design:**

Cohort study; Level of evidence, 2.

**Methods:**

A total of 870, excluding 9 players with a new contact ACL injury and a player with a new noncontact ACL injury just before the testing, female elite handball and soccer players—86 of whom had a history of ACL injury—underwent measurements of anthropometrics, alignment, joint laxity, and mobility, including leg length, knee alignment, knee anteroposterior laxity, generalized joint hypermobility, genu recurvatum, and hip anteversion. All ACL injuries among the tested players were recorded prospectively. Welch *t* tests and chi-square tests were used for comparison between the groups (new injury group, which sustained a new ACL injury in the follow-up period, and no new injury group).

**Results:**

An overall 64 new noncontact ACL injuries were registered. No differences were found between athletes with and without a new ACL injury among most of the measured variables. However, static knee valgus was significantly higher in the new injury group than in the no new injury group among all players (mean difference [MD], 0.9°; *P* = .007), and this tendency was greater in players with a previous ACL injury (MD, 2.1°; *P* = .002). Players with secondary injury also had a higher degree of knee hyperextension when compared with those previously injured who did not have a secondary injury (MD, 1.6°; *P* = .007).

**Conclusion:**

The anatomic factors that we investigated had a weak or no association with risk for an index noncontact ACL injury. Increased static knee valgus was associated with an increased risk for noncontact ACL injury, in particular for secondary injury. Furthermore, hyperextension of the knee was a risk factor for secondary ACL injury.

Anterior cruciate ligament (ACL) injury is one of the most severe injuries for athletes, especially in cutting sports such as soccer, handball, and basketball, as it leads to prolonged absence from competition and often suboptimal performance.^57,62,65^ Noncontact ACL injuries occur during cutting (change of direction) or 1-leg landing maneuvers,^30,41^ especially in female athletes. One study reported a secondary ACL injury rate of 20% for athletes who return to a sport.^67^ In other studies, patients with ACL reconstruction were at increased risk for the development of posttraumatic osteoarthritis and for knee arthroplasty in the mid- to long term.^8,33,64^ Noncontact ACL injury has multiple risk factors, which can be modifiable and nonmodifiable.^4,14^ Past studies have suggested various intrinsic factors, including high body mass, knee hyperextension, and anteroposterior (AP) laxity of the knee, as well as variations in knee anatomy, such as decreased ACL size, narrow intercondylar notch, increased posterior tibial slope, poor tibiofemoral congruity, and increased hip anteversion.^1,4,38,51,59,66^ Systematic review studies showed that generalized joint hypermobility in males and knee hyperextension increase ACL injury risk,^42,55^ although the association between generalized joint hypermobility and risk for ACL injury is still controversial in females.^55^ Moreover, injuries concomitant with ACL tears, such as medial collateral ligament injuries of grade ≥2,^56,68^ lateral meniscus posterior root tears,^24,44^ medial meniscus ramp lesions,^36,53^ and anterolateral structure injuries,^52,58^ are associated with residual rotatory laxity and have been shown to be risk factors for ACL failure, as well as younger age,^13^ increased posterior tibial slope,^16^ and knee hyperextension.^19^ Better understanding of risk factors, even nonmodifiable ones, is important to help inform players about risk and increase their understanding and motivation for injury prevention. Moreover, this information is crucial for orthopaedic surgeons to minimize failure risk after ACL reconstruction.

Unfortunately, our current knowledge is still limited. A prospective study analyzing the association between anatomic factors and noncontact ACL injuries in 859 cadets reported that primary ACL injury risk factors included small femoral notch width, generalized joint laxity, and, in women, higher body mass index and AP laxity of the knee. However, this study consisted of only 24 new noncontact ACL injuries, implying limited statistical power.^59^ To our knowledge, no other prospective studies have comprehensively investigated anatomic risk factors in female elite athletes. The purpose of this study was to prospectively investigate the relationship between anatomic factors (eg, anthropometrics, alignment, joint laxity, and mobility) and risk for future noncontact ACL injuries. Although we could add hypotheses—for example, that joint laxity (including AP laxity), hyperextension of the knee, and generalized joint hypermobility are risk factors for new ACL injury—the current investigation can be considered an explorative analysis that was not designed to test specific hypotheses.

## Methods

### Study Design and Participants

**Figure 1.**
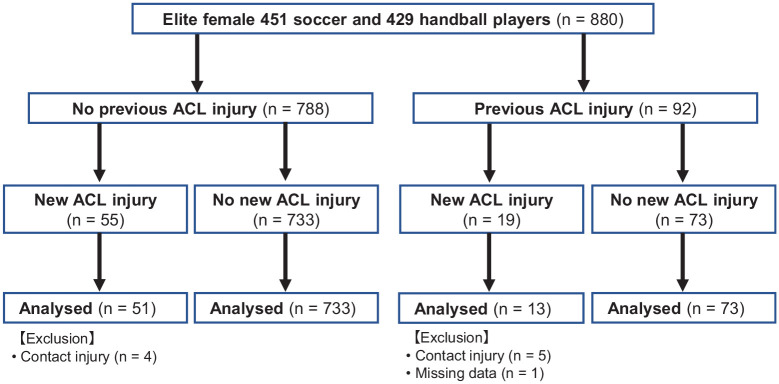
Flowchart detailing the study protocol. ACL, anterior cruciate ligament.

This prospective cohort study investigated the risk factors for noncontact ACL injuries in elite female handball and soccer players from 2007 to 2015. In 2007, all teams in the Norwegian female handball premier league were invited to a comprehensive preseason baseline screening examination. Athletes with a first team contract who were expected to play in the premier league during the 2007 season were eligible for participation. New teams qualified for the premier league from 2008 to 2013, and players from the clubs were invited for preseason tests. From 2009, female elite soccer players were also included. The tested sample represented approximately 84% of eligible athletes based on data from the first year of testing.^29^ In total, 880 players composed the cohort (handball, n = 429; soccer, n = 451; age, 14-37 years). Nine players with a new contact ACL injury were excluded. One player with a new noncontact ACL injury was excluded because she had no physical examinations owing to the ACL reconstruction that she had undergone 3 months before. Thus, 870 players were included in the analysis (Figure 1). Data were partly missing in 103 players for reasons such as ankle sprain sustained before the test, knee or Achilles pain, shortage of testing time, and problems with testing equipment. Demographic data such as age, mass, and height were collected at the time of a preseason screening examination. The Regional Committee for Medical Research Ethics, the South-Eastern Norway Regional Health Authority, and the Norwegian Social Science Data Services approved the study. All players gave informed consent before inclusion. Players aged <18 years provided parental consent.^31^

The primary outcome variables of this prospective cohort were defined by Krosshaug et al.^31^ All measurements and procedures were performed as described in our previous study^43^ and are therefore explained briefly.

### Anthropometrics and Alignment

#### Skin Marker–Based Measurements: Leg Length, Pelvic Orientation, and Knee Alignment

All reflective markers were attached on anatomic landmarks or muscle bellies: bilateral posterior and anterior superior iliac spines, greater trochanters, lateral and anterior thighs, lateral femoral condyles, tibial tubercles, anterior and lateral lower legs, lateral malleolus, heels, and laterally and medially near the tips on the shoes.^40^ From the static calibration trial, we defined each participant's natural posture and measured static knee valgus and varus alignment, femur and tibial length, and pelvis orientation (forward tilt, left tilt, left rotation) and width. For handball and soccer players, static alignment was measured while wearing their preferred indoor shoes.

The markers were used to estimate the participant's joint centers according to previous studies.^5,15,17^ The length of the femur and tibia was measured from the center of the hip joint to the knee joint and from the knee joint to the ankle joint, respectively. The total leg length was measured from the center of the hip joint to the floor. The lengths were presented as a ratio of total body height. Assessment of the pelvic orientation was performed with the test participant standing in a standard anatomic position. The orientation of the pelvis was measured relative to the global system. Pelvis width was defined as the distance between the right and left anterior superior iliac spines and presented as a ratio of total body height. Static knee valgus angle was measured as the frontal angle among the hip, knee, and ankle joint centers.

#### Femoral and Tibial Condyle Width

The femoral and tibial condyle width were measured between the medial and lateral condyles of the knee. The distance was measured to the nearest 0.5 cm, using a caliper. The width was presented as a ratio of total body height.

### Joint Laxity

#### Knee AP Laxity

The KT-1000 knee arthrometer was used to quantify the AP laxity of the knee as described previously.^40^ The maximum value (in millimeters) in each knee was recorded from 2 trials. The reliability of the KT-1000 arthrometer by experienced raters has been shown to be good (intraclass correlation coefficient, 0.79).^6^ Based on the International Knee Documentation Committee guidelines, normal knee AP laxity was defined as a side-to-side difference ≤2 mm.^21^

#### Genu Recurvatum

Genu recurvatum (hyperextension of the knee) was measured with a standardized goniometer. The testers palpated the anterior and posterior parts of the lateral knee joint line and marked the midpoint of the sagittal plane (K). The most prominent part of the lateral ankle malleolus (M) and greater trochanter (T) was then palpated and marked. The axis of the goniometer was positioned above each point, and the angle formed by the line K-M and the line K-T was measured and the nearest degree recorded. Plus values (≥0°) indicated genu recurvatum.

#### Generalized Joint Hypermobility

The Beighton score (total 9 points) was used to evaluate generalized joint hypermobility.^23^ Because of the analysis based on each leg, the Beighton score was calculated for each half of the body, and the forward touchdown was included for both halves of the body (score range, 0-5 points).

### Mobility

#### Hamstring Mobility

Testing of hamstring mobility was performed on an examination table with a firm surface and lumbar support. The player was lying on the bench in the supine position with the pelvis and the nontested leg stabilized using belts to avoid accessory movements. The hip of the testing leg was fixed at 120° of flexion using a belt, and the player was supported against further hip flexion by the tester pressing with both hands distally on the femur. The ankle and foot were relaxed, and the hip was in neutral rotation, abduction, and adduction. Three landmarks were placed on the leg: lateral fibular malleolus, lateral femoral epicondyle, and greater trochanter of femur. The knee was extended passively with an 8-kg load, and a goniometer was placed to the point of the knee joint line. Flexibility was measured as static range of motion.

#### Hip Anteversion

The player lay in a relaxed prone position on a therapy bench and was fixed with a belt over her pelvis. One test person held the distal part of the player's tibia, passively flexed the knee to 90°, and then rotated the hip passively inward and outward until the most lateral and prominent part of the greater trochanter was identified by palpation. In this position, another test person measured the angle between the vertical line and the shaft of the tibia to the nearest degree using a standardized goniometer.^48^

#### Navicular Drop

The navicular drop was defined as the distance that the navicular tuberosity moves in the standing position as the subtalar joint is allowed to move from its neutral position to a relaxed position. The height of the navicular tuberosity was measured in neutral and relaxed stance positions, and the total excursion was measured.^37^

### Injury Registration

All complete ACL injuries among the tested players were recorded through May 2015, primarily through semiannual contact with the participating teams (manager, coach, and medical staff). If any acute knee injuries occurring during regular team training or competition were reported, the injured player was contacted by phone to get detailed medical data and a description of the injury situation.^22^ Noncontact ACL injury, as defined in this study, also consisted of “indirect contact”: situations where player contact may have occurred before or at the time of injury but was not a direct blow to the knee (contact injury). All ACL injuries were confirmed by magnetic resonance imaging and/or arthroscopy.^31^

### Statistical Analysis

The data were analyzed using the statistical software R Version 4.2.2. Descriptive data are presented as mean and standard deviation. A total of 870 players were divided into 2 groups: no previous ACL injury (784 players) and previous ACL injury (86 players). We analyzed 18 anatomic measurements as described earlier. Players were divided into 2 subgroups: new injury group, which sustained a new ACL injury in the follow-up period, and no new injury group. Welch *t* test and a chi-square test were used for comparison between the groups. Statistical significance was set at *P* < .05.

## Results

Out of 880 players, 64 new noncontact ACL injuries were used in the analysis: 44 no contact and 20 indirect contact (Figure 1). Secondary ACL injuries occurred in 5 ipsilateral and 8 contralateral knees. The mean ± SD age at a new noncontact ACL injury during the study period was 21.9 ± 4.0 years. The mean duration from physical examination to a noncontact ACL injury was 1.9 ± 1.8 years. No player had a new noncontact ACL injury in both knees during the study period. Eight players had previous bilateral ACL injuries before testing.

**Figure 2.**
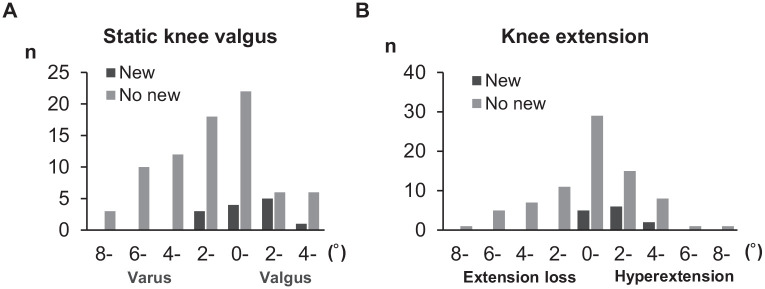
Distribution of (A) static knee valgus and (B) knee extension angle in players with previous anterior cruciate ligament (ACL) injury (n = 86).

Among the variables that were measured, we found only small or no differences between those who sustained a noncontact ACL injury and those who did not (Table 1). However, among all players, static knee valgus was significantly higher in the new injury group than in the no new injury group (0.2°± 2.6° vs −0.7°± 2.9°; *P* = .007), and this tendency was clearer in the previous ACL injury players (1.4°± 1.8° vs −0.7°± 2.9°; *P* = .002) (Figure 2A). Players with secondary injury also had a higher degree of genu recurvatum as compared with previously injured players who did not have a secondary injury (1.9°± 1.6° vs 0.3°± 3.0°; *P* = .007) (Figure 2B). No significant differences were found for KT-1000 arthrometer values and Beighton scores between groups with and without new injuries (Tables 1 and 2).

**Table 1:** Player Characteristics Classified According to Past and Current ACL Injury^*a*^

	All			No Previous Injury			Previous Injury		
	New Injury	No New Injury	*P* Value	New Injury	No New Injury	*P* Value	New Injury	No New Injury	*P* Value
**No. of players**	64	806		51	733		13	73	
Age at testing, y	20.3 ± 3.7	21.0 ± 4.0	.12	19.5 ± 3.3	20.7 ± 3.9	**.016**	23.2 ± 3.7	23.8 ± 4.1	.59
Body mass, kg	67.5 ± 8.0	66.1 ± 7.9	.17	66.3 ± 7.4	65.9 ± 7.9	.76	72.5 ± 8.6	67.6 ± 8.3	.07
Height, cm	170.6 ± 7.0	169.5 ± 6.3	.23	170.1 ± 6.8	169.3 ± 6.2	.43	172.3 ± 7.5	171.1 ± 6.9	.59
**No. of players (Range)^*b*^**	61-63	751-800		48-50	683-728		13	77-82	
Degrees (static)									
Knee valgus	0.2 ± 2.6	−0.7 ± 2.9	**.007**	−0.1 ± 2.7	−0.7 ± 2.9	.13	1.4 ± 1.8	−0.7 ± 2.9	**.002**
Pelvis forward tilt	15.0 ± 4.2	15.3 ± 4.6	.56	14.9 ± 4.3	15.5 ± 4.6	.34	15.6 ± 3.4	13.9 ± 4.1	.13
Pelvis left tilt	−0.4 ± 1.7	−0.5 ± 1.7	.80	−0.4 ± 1.8	−0.5 ± 1.7	.78	−0.4 ± 1.8	−0.4 ± 1.7	.85
Pelvis left rotation	0.7 ± 2.6	0.5 ± 2.8	.55	0.7 ± 2.7	0.5 ± 2.8	.55	0.7 ± 2.5	0.6 ± 2.8	.94
Percentage vs height									
Pelvis width	14.0 ± 1.0	13.9 ± 1.1	.25	14.0 ± 1.0	13.8 ± 1.1	.38	14.2 ± 0.9	14.1 ± 1.1	.58
Leg length	52.9 ± 1.7	52.4 ± 1.8	**.040**	52.9 ± 1.7	52.4 ± 1.8	.08	53.0 ± 1.9	52.5 ± 1.7	.34
Femur length	24.9 ± 0.9	24.8 ± 0.8	.39	25.0 ± 0.9	24.8 ± 0.8	.29	24.8 ± 0.7	24.8 ± 0.8	.87
Tibia length	24.5 ± 1.0	24.5 ± 1.0	.61	24.4 ± 1.1	24.5 ± 1.0	.65	24.5 ± 0.8	24.7 ± 1.0	.69
Femur condyle width	5.3 ± 0.7	5.4 ± 0.7	.58	5.3 ± 0.8	5.4 ± 0.7	.56	5.3 ± 0.6	5.3 ± 0.7	.59
Tibia condyle width	5.1 ± 0.6	5.2 ± 0.6	.60	5.1 ± 0.6	5.2 ± 0.6	.66	5.1 ± 0.5	5.0 ± 0.6	.69
KT-1000, mm	7.0 ± 2.0	6.9 ± 1.9	.71	6.8 ± 1.9	6.8 ± 1.8	.99	7.8 ± 2.2	8.4 ± 2.6	.37
Genu recurvatum, deg	0.6 ± 3.1	1.0 ± 3.5	.46	0.3 ± 3.3	1.0 ± 3.5	.16	1.9 ± 1.6	0.3 ± 3.0	**.006**
Beighton score	1.1 ± 1.2	0.9 ± 1.0	.44	1.1 ± 1.3	0.9 ± 1.0	.22	0.8 ± 0.9	1.2 ± 1.1	.14
Hamstring mobility, deg	137 ± 12	139 ± 12	.38	137 ± 12	138 ± 12	.48	139 ± 9	143 ± 12	.12
Hip anteversion, deg	7.9 ± 4.6	9.4 ± 5.0	**.014**	8.0 ± 4.1	9.4 ± 5.0	**.020**	7.6 ± 6.2	9.4 ± 5.2	.35
Navicular drop, mm	50.2 ± 5.8	49.6 ± 4.7	.42	50.2 ± 5.9	49.6 ± 4.8	.45	50.3 ± 5.9	49.9 ± 4.7	.83

a Data are presented as mean ± SD. *P* values are shown for univariate comparisons between players with and without a new noncontact ACL injury. Bold indicates *P* < .05. For players with new injuries, data are reported for the injured leg only (except age, body mass, and height). In the groups with no new injuries, data represent the mean of the left and right legs, although data among the players with previous injury represent the mean of the all previously injured legs. ACL, anterior cruciate ligament.

b The number of tested players varied by test.

**Table 2:** SSDs of KT-1000 Arthrometer Values According to Previous and Current ACL Injury^*a*^

	All			No Previous Injury			Previous Injury		
	New Injury	No New Injury	*P* Value	New Injury	No New Injury	*P* Value	New Injury; I/C	No New Injury	*P* Value
SSD, mm			.90			.80			.56
≤2	52 (82.5)	667 (83.8)		44 (88.0)	630 (86.8)		8 (61.5); 2/6	37 (52.9)	
>2	11 (17.5)	129 (16.2)		6 (12.0)	96 (13.2)		5 (38.5); 3/2	33 (47.1)	

a Data are presented as No. (%) of players. *P* values are shown for univariate comparisons between players with and without a new noncontact ACL injury. For all groups, the data represent SSD regardless of previous or new injury. ACL, anterior cruciate ligament; I/C, ipsilateral knee/contralateral knee; SSD, side-to-side difference.

Among all players, the new injury group had a significantly lower hip anteversion (7.9°± 4.6° vs 9.4°± 5.0°; *P* = .014) than the no new injury group, as did players with no previous injury (8.0°± 4.1° vs 9.4°± 5.0°; *P* = .020). Although a small difference among all players, leg length relative to height was significantly larger in the new injury group than the no new injury group (52.9% ± 1.7% vs 52.4% ± 1.8%; *P* = .040). Among the players with no previous ACL injury, those with a new ACL injury were significantly younger than those with no new ACL injury (19.5 ± 3.3 vs 20.7 ± 3.9 years; *P* = .016) (Table 1).

## Discussion

The present study revealed that only a few anatomic risk factors were associated with an ACL tear in elite female athletes. AP laxity of the knee and generalized joint laxity were not found to be risk factors for either primary or secondary ACL injury. However, increased static knee valgus was associated with an increased risk for primary and secondary ACL injury, suggesting that noncontact ACL injury risk is related not only to movement patterns such as knee valgus and knee abduction moment at cutting/landing but also to frontal knee alignment. Furthermore, hyperextension of the knee was associated with an increased risk for secondary injury. To our best knowledge, the current study is the first prospective study to evaluate the association between secondary ACL injury and anatomic risk factors in elite female athletes.

Previous studies^27,34^ suggested that frontal plane biomechanics plays an important role for noncontact ACL injury risk in sports. Results from the current study indicate that injury risk is related not only to movement patterns of high knee abduction moment at cutting/landing but also to anatomic lower limb alignment of the players. The static knee valgus angle was significantly greater in the new injury group than in the no new injury group for all players, but the difference was mainly driven by the players with a history of ACL injury (Table 1). This suggests that special attention should be given to ensure good knee alignment before and after surgery—for example, through appropriate treatment of concomitant injury that may affect valgus alignment, such as medial collateral ligament injury^35^ and lateral meniscus tear,^20^ and neuromuscular rehabilitation. However, it should be pointed out that we have no indications for unsuccessful surgery in players with secondary injury, because the group with no previously injured leg had slightly higher valgus (0.88°± 2.2°) than the group with a previously injured leg (0.69°± 3.0°). Nevertheless, the current study is clinically relevant because it implies that noncontact ACL injury risk is related not only to dynamic valgus in cutting/landing situations but also to frontal knee alignment.

Hyperextension of the knee (defined as genu recurvatum >0°) was not a risk factor for primary ACL injury in our cohort of elite female athletes (Table 1). This finding stands in contrast to previous studies that have suggested knee hyperextension to be a risk of primary ACL injury.^28,38,46^ However, these studies either had smaller sample sizes as compared with the current study or were case-control studies with possible biases. Although the heterogeneous definition of hyperextension (recurvatum >0°, >5°, or >10°)^11,12,18,38,46^ should be taken into account when discussing studies on knee hyperextension, our study has far greater statistical power than previous studies. In contrast to primary ACL injury, knee hyperextension >0° was identified as a risk factor for secondary ACL injury, including new injury to the contralateral side (n = 8) in addition to graft rupture (n = 5). Furthermore, in recent studies, preoperative knee hyperextension >5° and >6.5° was identified as a risk factor for ACL hamstring tendon graft failure.^18,19^ These authors measured passive knee hyperextension in the contralateral (healthy) knee at the time of the surgical procedure and under anesthesia, which may have contributed to the differences in the measured knee hyperextension between these studies and the present study. Therefore, it may be necessary to pay special attention to the presence of knee hyperextension in athletes with a history of ACL injury. Yet, it should be noted that the small sample size in our cohort in the analysis of secondary ACL injuries results in low statistical power.

Several studies have evaluated the association between primary ACL injury and AP laxity. Vacek et al^60^ reported that for each additional millimeter in knee AP displacement present, there was 27% greater odds of sustaining an ACL injury (odds ratio, 1.27) among young female athletes. In contrast, in a prospective study, Uhorchak et al^59^ observed no statistically significant difference in mean KT-2000 arthrometer results between young female cadets with and without an ACL injury. Shimozaki et al^50^ and Vauhnik et al^63^ also showed no significant difference of knee anterior laxity between female athletes with and without ACL injury. Taken with the results of the current study (Table 1), AP laxity of the knee is unlikely to be a risk factor for primary ACL injury among young female athletes. Moreover, focusing only on those with AP laxity >2 mm, which is considered abnormal knee laxity,^21^ did not change the outcome of the current study, as the proportion of players with high AP laxity was not increased in players with a new injury (Table 2).

In contrast to 2 previous studies, neither AP laxity (Table 1) nor side-to-side difference in AP laxity of the knee (Table 2) was found to be a risk factor for secondary ACL injury. Pinczewski et al^45^ reported a side-to-side difference in AP laxity of >2 mm at 2 years after surgery to be associated with an increased risk for graft rupture in a 10-year follow-up study after ACL reconstructions. Moreover, Bourke et al^9^ reported that patients with a side-to-side difference of AP laxity >2 mm at 1 year postoperatively had a 2.9-fold risk of ACL retear at 15-year follow-up after ACL reconstruction. The discrepancy between the results of the aforementioned 2 studies and the present study might have originated from the fact that ACL secondary injuries in our study also include contralateral ACL injuries. In a further breakdown of the current results, the proportion of players with ACL reinjury and a side-to-side difference of AP laxity >2 mm was higher than that of players with no new ACL injury and a side-to-side difference >2 mm (60.0% [3/5] vs 44.8% [35/78]); however, in the current study, the limited number of reinjuries in the material prevents conclusive interpretation.

In the current study, generalized joint hypermobility was not a risk factor for primary or secondary ACL injury. A number of studies have suggested an association between generalized joint hypermobility and ACL injury.^2,25,46,54,61^ However, among only prospective studies on women, 1 study reported generalized joint hypermobility as a risk for primary ACL injury,^59^ while 2 studies found no significant association.^51,60^ As for secondary ACL injury, 1 prospective and 2 retrospective studies showed a higher incidence of either ACL graft rupture and contralateral ACL injury or ACL failure in patients with generalized joint hypermobility than in patients without generalized joint hypermobility.^25,26,32^ Yet, in the present study, there was not even a tendency suggesting that there might be a difference between those with and without new noncontact ACL injury regardless of previous ACL injury; rather, the Beighton score, which is a reliable clinical assessment tool that shows acceptable reliability when used by raters of any background or experience level,^7^ tended to be slightly higher in the no new injury group. Even if the Beighton score was composed of the left and right sides to give a maximum score of 9, the mean Beighton score for the new injury group in the whole cohort would still be low at 2.2. It should be considered that the proportion of females with generalized joint laxity (Beighton score ≥4) may be lower in this study of elite female athletes than in the general female population (23% vs 51.0%, respectively).^10,47,49^ The present prospective study has a larger sample size of female elite players and higher statistical power than previous studies. Therefore, our study revealed that generalized joint laxity is not a risk factor for ACL injury, at least among young elite female athletes in cutting sports.

The current study did not support a hypothesis from a previous study based on magnetic resonance imaging measurement suggesting that increased hip anteversion would increase ACL injury risk.^1^ We found hip anteversion to be significantly smaller in players sustaining a new injury among players without a previous injury. There was a similar tendency among players with a previous ACL injury (Table 1). A prospective cohort study of 290 female high school basketball and handball players reported similar results (noncontact ACL group [15.3°] vs control group [16.6°]) and suggested that reduced femoral anteversion could be a risk factor for noncontact ACL injury.^39^ Although we observed the same trend, the mean difference in hip anteversion between groups was only 1.5°, which indicates limited usefulness in clinical practice considering the measurement error. Given the error-prone nature of hip anteversion measurement, the association between hip anteversion and ACL injury risk is still controversial, and future studies based on more accurate testing methods will be needed.

Players who sustained a new ACL injury had longer leg length relative to their height as compared with the injury-free players (Table 1). Longer leg length is likely to result in higher external moment arms and may thereby contribute to increased knee abduction moment. However, the observed differences in leg length between players with and without a new injury corresponded to <1 cm and is therefore likely of limited clinical significance.

The present study has unique strengths when compared with previous risk factor studies because of the large sample size and homogeneous participants, including only female elite athletes. However, there are some limitations in the present study. First, errors in physical examination measurements and marker placements could have occurred. To minimize these errors, we used the same clinician for multiple testing years. Nevertheless, there were still several testers over the 8-year data collection period. In our reliability test conducted on 106 players in the same cohort with a 2-year interval, mean ± SD differences for knee static angle, genu recurvatum, and hip anteversion were −1.6°± 3.2°, 0.3°± 3.2°, and 8.6°± 3.7°, respectively. Second, some athletes might still be growing during the follow-up period, but with an average age of 21 years, this will likely have limited influence on our data. Third, follow-up periods varied among all players, and playing exposure times were not collected. Yet, as the percentage of players with a new injury in the cohort was as low as 7.9%, Cox regression analyses are not warranted.^3,31^ Fourth, we decided to do simple univariate analyses in the current study, rather than investigating odds ratios using logistic regressions. We made this decision as the study power will not allow for the large number of outcome variables and possible adjustment factors. We had already conducted advanced machine learning analyses that showed poor predictive ability of future noncontact ACL injury, even when utilizing far more tests and variables among the 791 players in the current cohort.^22^ Fifth, no radiologic analysis including femoral notch size and proximal tibial slope was performed, as this was not practically possible in this data collection. Last, no detailed information was available on ACL reconstruction, including graft choice and surgical technique.

## Conclusion

Most anatomic factors, including anthropometrics, joint laxity, alignment, and mobility, had a weak or no association with risk for primary noncontact ACL injury in elite female athletes. However, increased static knee valgus was associated with an increased risk for noncontact ACL injury, in particular for secondary injury. Furthermore, hyperextension of the knee was identified as an additional risk factor for secondary ACL injury. Yet, the number of players with secondary injury was limited, meaning that these results must be treated with caution.
